# Vitamin D status of northern indigenous people of Russia leading traditional and “modernized” way of life

**DOI:** 10.3402/ijch.v73.26038

**Published:** 2014-12-02

**Authors:** Andrew Kozlov, Yulia Khabarova, Galina Vershubsky, Yulia Ateeva, Vadim Ryzhaenkov

**Affiliations:** 1 Institute and Museum of Anthropology, Moscow State University, Moscow, Russia; 2 Research Department, Perm State Humanitarian Pedagogical University, Perm, Russia; 3 Department of Family Medicine, Northern State Medical University, Arkhangelsk, Russia; 4 Federal Center for Hygiene and Epidemiology, Perm, Russia

**Keywords:** 25OHD, nutrition, lifestyle, reindeer herders, circumpolar regions, Arctic

## Abstract

**Background:**

Vitamin D status in groups of northern indigenous people of Russia leading close to traditional (seminomadic reindeer herding), post-traditional (in settlements) or “modernized” (in towns) way of life was analysed.

**Design:**

The survey study groups consisted of 178 Nenets and Komi aged 18–60 living in the Arctic (66–67°N). Urban Komi, Udmurts and Komi-Permiaks (n=150) living in a non-Arctic area (57–61°N) formed a control group. The concentration of serum 25-hydroxyvitamin D (25OHD), as a transport form of vitamin D, was assessed by enzyme immunoassay analysis.

**Results:**

The group average 25OHD levels in both rural and urban Arctic residents are within the range of values seen in the non-Arctic urban subjects adjusted for season: 39.7–47.7 nmol/l. Abandoning traditional lifestyle associates with lower vitamin D levels in indigenous Arctic people. Mean±standard deviation 25OHD values among Nenets were lower in those living in the administrative centre (a big settlement) with a population of 1,460 (32.2±12.90 nmol/l) than in the residents of small settlements (39.6±14.08 nmol/l), and in reindeer herders (42.4±13.45 nmol/l; p<0.05 in both cases). Komi townspeople had lower 25OHD concentrations (47.7±12.00 nmol/l) than Komi reindeer herders (68.7±25.20; p<0.01).

**Conclusions:**

The transition from seminomadic to post-traditional and “modernized” way of life has led to a decrease in the consumption of traditional foods among the indigenous people of the Russian Arctic. Our data support the notion that the traditional northern diet promotes healthy vitamin D levels, while adherence to the “western” type of diet correlates with a lower 25OHD concentration.

The term “vitamin D” designates two steroid prohormones of different origin. Ergocalciferol (D_2_) is produced from plant sterols provided by the diet. Cholecalciferol (D_3_) is synthesized in the skin under exposure to ultraviolet radiation. Both substances are converted into 25-hydroxyvitamin D (25OHD) in the liver. Serum concentration of 25OHD reflects vitamin D produced cutaneously and that obtained from food and supplements. Circulating in the bloodstream 25OHD is the transport form of the vitamin and its main reservoir in the human body. Vitamin D deficiency hinders intestinal absorption of calcium and phosphorus, and renal reabsorption of these minerals. The change in bone tissue due to vitamin D deficiency clinically manifests itself as rickets in children, and osteomalacia and osteoporosis in adults (1).

Nowadays, high-latitude regions are regarded as an endemic risk area for vitamin D deficiency (2). However, in pre-Columbian North America populations bone tissue metabolism disorders seem to be rare (3). Furthermore, scholars report that modern Inuit and Amerindians even having relatively low vitamin D levels show satisfactory characteristics of bone mineral metabolism and bone mass (4–6). The lower levels of 25OHD of modern Native Americans as compared with Europeans and Euro-Americans could be an ethnic-specific norm. In this case, there is no particular reason for concern. If the relatively low vitamin D levels in indigenous northerners are a consequence of “modernizing” changes, then additional measures to prevent rickets and osteoporosis are necessary (6).

A comparison of serum 25OHD levels in populations living in the same geographic region, but having different types of diet – either “westernized” or close to traditional – can shed light on this issue.

In our study, we examine the indigenous northerners of Russia. In the last decade of the 20th century, many of them, for economic and social reasons, reverted to a traditional subsistence economy and use mostly local produce (7). The transition resulted in a situation where representatives of an ethnic group living in one geographic region have different dietary schemes. Townspeople almost completely depend on commercial or store-bought foods. Settled residents of northern villages combine store-bought and local foods. The diet of reindeer herders is close to traditional and based mainly on the harvest of herds. Reindeer herders are seminomadic, in that they work in shifts spending some time (usually 10–15 days) with herds in the tundra and then return to the village for the same term.

Comparing the serum 25OHD levels of these groups can provide information about the role of a traditional diet in maintaining the D-vitamin status of northern people.

## Objective

The study aims to compare levels of serum 25OHD and vitamin D status of northern indigenous people of European Russia in populations leading close to traditional, post-traditional, and “modernized” ways of life.

## Participants and methods

### Participants

A total of 328 blood samples were collected in 2009–2012 in several Arctic and northern population groups of the Russian Federation. The simple random sampling formed all the study groups during annual preventive medical check-ups, which took 2–10 days at every locality. The length of day on the middle dates of sample collection was calculated for every geographic point using *http://www.timezone.ru/suncalc.php* tool. Subjects were aged 18–60. Ethnicity was self-reported. The geographical locations of herding and camping grounds at the times concurrent with the sample collection are quoted from the report of the Norwegian Polar Institute/Association of Nenets People “Yasavey” (8). The main characteristics of the study groups are given in Table I.

**Table I:** Characteristics of study groups

Residency name	Residency type; population	Ethnic group, n	Age	Geogr. latitude (°N)^a^	Day length (hh:mm), date
Nes	Village; 1,460	Nenets, 42	18–59	66°36′	02:42, Dec 10, 2012
	Camps	Nenets, 40	21–60	67°00′	02:38, Dec 10, 2012
Khorey-Ver	Village; 740	Nenets, 46	20–58	67°24′	01:20, Dec 10, 2012
	Camps	Nenets, 37	18–56	68°00′	00:00, Dec 10, 2012
Izhma	Camps	Komi, 13	18–52	66°00′	08:30, Feb 10, 2010
Syktyvkar	City; 240,000	Komi, 52	17–23	61°21′	07:35, Nov 10, 2009
Izhevsk	City; 633,000	Udmurt, 52	21–58	56°51′	08:10, Nov 15, 2011
Kudymkar	Town; 35,000	Komi-Permiak, 46	19–59	59°01′	10:31, Mar 1, 2010

a For Camps it is average on the date.

The indigenous people of the Arctic regions of the Russian Federation in our study were represented by two ethnic groups.

The first was the Nenets from the Nenets Autonomous Okrug (NAO). The serum was collected in wintertime (December). The subjects were residents of Nes (the administrative centre of Kaninskiy district of the NAO) and Khorey-Ver. Samples were taken from settled residents and reindeer herders spending considerable time in the tundra with their herds. The reindeer herders’ samples were collected at a time when seasonal grazing areas and temporary camps were relatively close to the villages. Analysing nutrition in Nenets, we used the data published by Murashko and Dallmann (8). They studied the diet of the indigenous people of the NAO in 2007–2009 and, in particular, collected data in Nes and Khorey-Ver villages.

The second ethnic group was the Komi living in Izhma village (Izhemsky district of the Republic of Komi). They were examined in late winter (February). Like the Nenets, the Komi reindeer herders are seminomadic and herd their stock in the polar tundra on the territory of the NAO. Data on vitamin D status in the Komi have been published previously (9), but this study used other diagnostic criteria, and the results partially differ from those published.

A reference group consisted of urban inhabitants of non-Arctic regions of the European part of the Russian Federation – ethnic Komi, Udmurts and Komi-Permiaks. These ethnic groups belong to the Eastern-Finnish peoples. The Komi living in Syktyvkar city and the Udmurts of Izhevsk city were examined in autumn (November). The samples from the Komi-Permiak residents of Kudymkar town were collected in spring (March).

### Ethics

Local health authorities approved the conduct of the study. All participants gave written consent to participate in it upon being informed about the methods and goals of the examination. Consent forms were composed in Russian, in which all the participants were articulate.

### Clinical assessments

Blood was drawn from the cubital vein into “Becton Dickinson BD” (England) vacutainers. Serum was prepared by centrifugation of the blood at 3,000 rpm for 15 minutes and freezing for 2–3 hours and was stored at −30°C until analysis.

### Laboratory analysis

The blood serum concentration of the transport form of vitamin D – 25OHD – was determined by enzyme immunoassay analysis using the “Immunodiagnostic Systems Ltd” (Boldon, UK) kits (IDS EIA, standardized manual procedure). According to current recommendations (10), we regarded the serum 25OHD concentrations of 30 and 50 nmol/l as boundaries between the deficient (prominent hypovitaminosis), insufficient (marginal hypovitaminosis) and sufficient vitamin D statuses.

### Statistical analysis

Preliminary analysis showed no sex differences in serum 25OHD in any of the study groups. They are also quite uniform by age composition. Hence, there was no subdivision by age and sex in further analysis. A logarithmic transformation was applied on individual 25OHD values to make the distribution more symmetrical. Log transformed 25OHD levels were used in cross-group comparisons by the nonparametric Mann-Whitney U test. We applied the Pearson's chi-square test to estimate the differences in vitamin D status distributions between the groups.

## Results

Table II depicts the descriptive statistics of the data. Among the settled rural Nenets, the serum 25OHD content was lower in the residents of Nes than Khorey-Ver village (p<0.05). The vitamin D status prevalence also differed significantly in this sample groups (p<0.0001). Almost half (45%) of Khorey-Ver residents were vitamin D sufficient and only 7% had prominent hypovitaminosis. Among Nes residents 13% were sufficient and 53% deficient (Table II).

**Table II:** Serum 25OHD concentrations in study groups (adults, both sexes combined)

			25OHD (nmol/L)			Vitamin D status (%)		
				
Ethnic group	Residency	Way of life	n	M	SD	SF	IS	DF
Nenets	Nes	Rural	42	31.3	12.72	13	34	53
		Seminomadic	40	35.3	11.33	8	58	33
Nenets	Khorey-Ver	Rural	46	47.1	10.64	45	48	7
		Seminomadic	37	50.2	11.12	51	43	6
Komi	Izhma	Seminomadic	13	68.7	25.20	84	8	8
Komi	Syktyvkar	Urban	52	47.7	12.00	41	55	4
Udmurts	Izhevsk	Urban	52	44.6	12.90	31	56	13
Komi-Permiak	Kudymkar	Urban	46	44.7	9.00	28	70	2

SF=sufficient, IS=insufficient, DF=deficient.

In the combined (both the villages) group, 25OHD levels were significantly higher in the seminomadic reindeer herders (n=77) than in the rural settled Nenets (n=88; p<0.05). However, the situation was different within each village. The differences between the seminomadic and the settled Nenets were statistically significant in Khorey-Ver (p<0.01), but not in Nes (p=0.09). Among the seminomadic residents, the number of vitamin D sufficient subjects was considerably higher, and the number of deficient subjects lower, in Khorey-Ver than in Nes (p<0.0001).

The ethnic Komi seminomadic reindeer herders of Izhma had the highest 25OHD levels among all the study groups. The difference is significant when comparing the Komi group with the seminomadic Nenets groups of both Nes (p<0.001) and Khorey-Ver (p<0.05). There were one case of deficiency and one case of insufficiency found in the group of 13 Komi reindeer herders. The differences in the prevalence of vitamin D statuses are also significant when comparing the seminomadic group of Izhma with both the Nenets groups (p<0.05).

The Komi reindeer herders of Izhma had significantly higher levels of 25OHD than the Komi, Komi-Permiaks and Udmurts living in urban conditions (p<0.01 in all cases). The 25OHD levels of the seminomadic Nenets of Khorey-Ver were significantly higher than in the urban Komi-Permiaks and Udmurts (p<0.05), and insignificantly higher than in the Komi of Syktyvkar city. The Nenets of Nes, both settled and seminomadic, showed lower vitamin D content as compared with all the reference groups of urban residents (p<0.001).

## Discussion

The vitamin D status of the population of Russia is poorly studied, so there is not much data for our materials on the native northerners to compare with. As far as we know, the only research devoted to vitamin D status in the Natives of the Russian Arctic was conducted in the NAO (11).

Our data show that the 25OHD levels in the urban Komi of Syktyvkar do not differ from that in the other study groups of the European part of Russia – the Eastern Finns (Komi-Permiaks and Udmurts – Table II), as well as Karelians (12), and the Russians (13–15). On average, settled rural Nenets have 25OHD concentrations within the range of the values found in urban ethnic Karelians and Russians. It seems that the 25OHD levels of settled rural and urban native northerners of the Russian Federation in general do not differ from that in non-Aboriginal populations, or, at least, these differences are small. This is in contrast to the situation described in various groups of the Canadian population. As Weiler et al. (16) reported, average serum 25OHD concentrations were 39.6±12.4 nmol/l in rural Aboriginal Canadian women 25–50 years old, 50.7±25.4 nmol/l in urban Aboriginals, and 66.6±34.9 nmol/l in urban white females.

According to our data, inhabitation of high latitude by itself does not negatively influence the vitamin D status of a population. It is clearly seen that the Komi reindeer herders have high 25OHD values, while the Nenets (without regard to their residence and occupation) are within the range of other ethnic groups living further to the south, at latitudes 57–61 north (Table II).

**Fig. 1:**
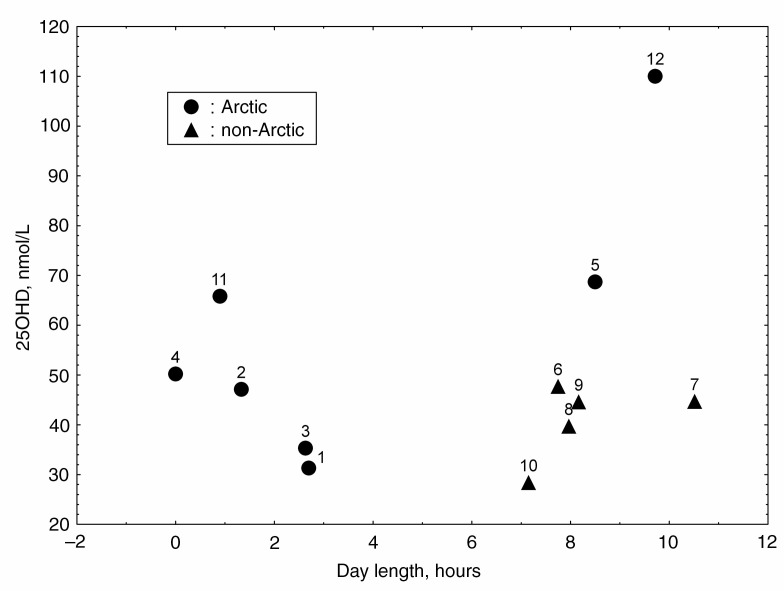
Serum 25OHD concentrations in various ethnic groups of Russia according to length of day (adults). 1 – Nenets, rural, Nes village; 2 – Nenets, rural, Khorey-Ver village; 3 – Nenets, seminomadic, Nes; 4 – Nenets, seminomadic, Khorey-Ver; 5 – Komi, seminomadic, Izhma village; 6 – Komi, Syktyvkar city; 7 – Komi-Permiaks, Kudymkar town; 8 – Russians, Perm urban agglomeration (14); 9 – Udmurtians, Izhevsk city; 10 – Karelians, Petrozavodsk city (11); 11 – Nenets, Naryan-Mar town (10); 12 – Nenets, seminomadic, Varandey and Varnek villages (10).

It is difficult to estimate the relation between the serum 25OHD concentration and the length of daytime judging only by the data collected by our research team. All the study groups of Nenets were examined in wintertime, when day length is minimal (0–3 hours). The Komi reindeer herders were surveyed under conditions of longer daytime (8½ hours). They have higher 25OHD levels than the seminomadic Nenets (p<0.05, Table II, Fig. 1). This difference may be attributed to UV radiation, as well as to other factors, such as cultural and race belonging.

To illustrate the suggestion that level of natural light influences vitamin D status at high latitudes, we use the data published by Blazheevich et al. (11). The authors reported the 25OHD levels measured in urban Nenets in winter (in December, when the daytime was less than 1 hour) and in Nenets reindeer herders in March (when the daytime was about 10 hours). It is seen on the diagram (Fig. 1) that the vitamin D content in the serum of Nenets (sample groups 11 vs. 12), and of Nenets and Komi reindeer herders (sample groups 3 and 4 vs. 5) taken at different length of day exhibits the same tendency: the longer the day hours, the higher the 25OHD concentrations. Thus, the available materials do not contradict the notion that UV radiation does influence the vitamin D status of the Arctic aborigines (17, 18).

**Fig. 2:**
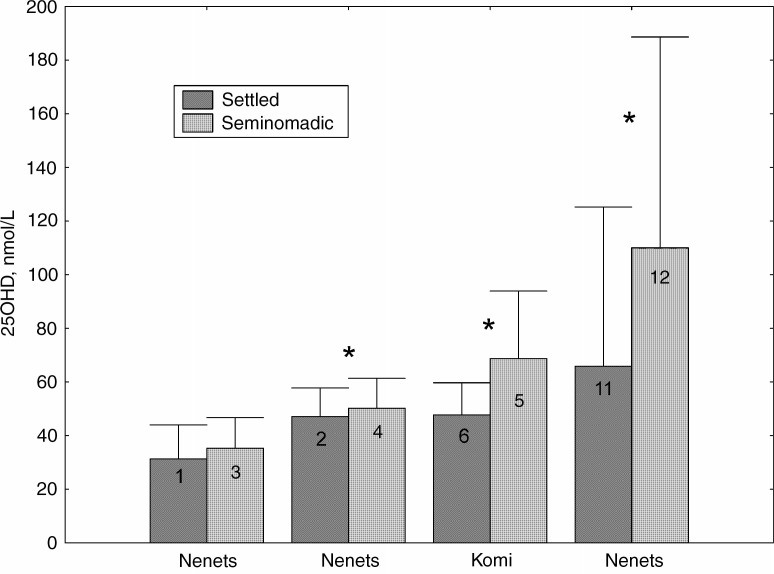
Serum 25OHD concentrations in settled and seminomadic groups in circumpolar and northern regions of Russia (adults). *p< 0.05. Legend as in Fig. 1.

With more confidence we can interpret the data concerning the relation between 25OHD content and the way of life and diet of northerners. In all the cases, 25OHD levels were higher in the seminomadic reindeer herders than in both the settled rural and urban Nenets, and the urban Komi (Fig. 2). In other words, the indigenous northerners of Russia leading a close to traditional lifestyle had, on average, a better vitamin D status than those living in villages and towns.

Rural dwellers in Russia are mainly oriented toward home grown or harvested produce. The consumption of self-provisioned meat in 2010 was 1.7 kg per capita per year in the urban populace, while in rural residents it was 10 times higher −16.6 kg (19). In the Russian Arctic, the share of local foods in the diet is particularly high. Self-harvested products constitute 68% of the diet of settled Nenets villagers of the NAO, and 84% of the diet of reindeer herders of Khorey-Ver (8).

The produce of reindeer husbandry forms the basis of the diet of Nenets and Komi reindeer herders. Reindeer breeding provides 30–40% of the food supply for the settled rural Nenets of NAO, and about 70% for the reindeer herders of Khorey-Ver (8). It is essential in the vitamin D context, since the fat of reindeer (*Rangifer tarandus*), unlike that of other animals, contains significant amounts of vitamin D (20). A human, being a higher-order consumer, gets significant amount of vitamin D from venison.

In addition to reindeer meat and fat, a significant contribution to the “Arctic diet” typical for the Nenets and Komi reindeer herders is made by fish (21). In the NAO, the per capita consumption of fish is 30% higher than in the Komi Republic, and twice as high as in the Komi-Permiak AO (9). Fishery products cover 30–40% of the food requirements of the settled rural Nenets in the NAO, and about 15–20% of that of the reindeer herders of Khorey-Ver (8).

It should be pointed out, however, that the Komi and Nenets villagers of Nes and Khorey-Ver mainly use freshwater fish species, which have lower vitamin D content than marine fish do. The small groups of Nenets residing at the seaside practiced sea-fishing and hunting along with reindeer breeding. For example, this was observed in Varnek settlement on Vaygach Island and in Varandey settlement on the Barents Sea (8, 21). At present, these villages do not exist: Varandey was closed up officially in 2000, and there are only 100 inhabitants left in Varnek. However, when in the early 1980s Blazheevich et al. (11) examined the residents of these settlements, their diet included the products of marine fishery. This explained, presumably, the very high levels of 25OHD in this sample group (Fig. 2).

The transition from the nomadic or seminomadic to post-traditional (in villages) and modernized (in towns) way of life resulted in a reduction of the share of traditional products in the diet of indigenous people in the Russian Arctic (7). It was also accompanied by a decrease in the levels of serum 25OHD. The gap between the study groups of tundra reindeer herders and Komi and Nenets townspeople is the most revealing (Fig. 2 – groups 5–6, and 11–12).

The settled rural residents also have lower 25OHD levels than the seminomadic herders, but the difference is less prominent (Fig. 2 – groups 1–3, and 2–4). Similarities in the diet and lifestyle, as well as close intra-community relations justify this. In the post-Soviet era the economically deprived people of the North returned to the once traditional foraging – fishing and hunting. It is also important that there is a common northern tradition of giving harvested goods to the unrelated poor and elderly (21). All of these factors diminish differences in the diet between the seminomadic and settled Nenets villagers and affect the vitamin D status in these groups.

Our data show that the traditional diet of northerners associates with a higher vitamin D status, but the transition to a “westernized” diet is associated with a decline in serum 25OHD concentrations. This is consistent with the findings of other researchers from Russia (11), Greenland (22, 23) and Canada (16, 24) (25).

## Conclusions

Rural and urban indigenous northerners in the Russian Federation do not differ significantly from non-indigenous population groups by the levels of serum 25OHD. Residency in high-latitude regions by itself does not impair the vitamin D status of a population.

The available data do not contradict the notion that serum 25OHD concentrations of the indigenous people of the North correlate with the length of daytime.

In Russia, within an indigenous northern ethnic group, those leading a close to traditional lifestyle, in general, have higher vitamin D status than the settled villagers and townspeople. The transition from nomadic or seminomadic to settled post-traditional (in villages) and modernized (in towns) life leads to a reduction of the share of local and traditional foods in the diet of indigenous people of the Russian Arctic. It is also accompanied by a decrease in the levels of serum 25OHD.

The traditional diet of inland Nenets and Komi indigenous Arctic people, which includes large amount of venison, reindeer fat, and fish, effectively prevents hypovitaminosis D.

## Supplementary Material

Vitamin D status of northern indigenous people of Russia leading traditional and “modernized” way of life
