# Supplementary material for: Vitamin D status of northern indigenous people of Russia leading traditional and “modernized” way of life
Source: Int J Circumpolar Health. 2014 Dec 2;73:10.3402/ijch.v73.26038. doi: 10.3402/ijch.v73.26038 (PMC4255095; doi:10.3402/ijch.v73.26038)
Supplement: Vitamin D status of northern indigenous people of Russia leading traditional and “modernized” way of life [file IJCH-73-26038-s001.pdf]

SUPPLEMENTARY FIGURES

Figure 1. Serum 25OHD concentrations in various ethnic groups of Russia according to length of day (adults).

Group	Day length (hh:mm)	25(OH)D, nmol/L	Arctic/ non-Arctic
1	02:42	31.3	Arctic
2	01:20	47.1	Arctic
3	02:38	35.3	Arctic
4	00:00	50.2	Arctic
5	08:30	68.7	Arctic
6	07:45	47.7	non-Arctic
7	10:31	44.7	non-Arctic
8	07:58	39.71	non-Arctic
9	08:10	44.60	non-Arctic
10	07:09	28.4	non-Arctic
11	00:54	65.8	Arctic
12	09:43	110.0	Arctic

Legend: 1 – Nenets, rural, Nes village; 2 – Nenets, rural, Khorey-Ver village; 3 – Nenets, seminomadic, Nes; 4 – Nenets, seminomadic, Khorey-Ver; 5 – Komi, seminomadic, Izhma village; 6 – Komi, Syktyvkar city; 7 – Komi-Permiaks, Kudymkar town; 8 – Russians, Perm urban agglomeration (14); 9 – Udmurts, Izhevsk city; 10 – Karelians, Petrozavodsk city (11); 11 – Nenets, Naryan-Mar town (10); 12 – Nenets, seminomadic, Varandey and Varnek villages (10).

Figure 2. Serum 25OHD concentrations in settled and seminomadic groups in circumpolar and northern regions of Russia (adults).

Group	Settled / Seminomadic	25OHD, nmol/L			p
		N	M	SD	
1	Settled	42	31.3	12.72	NS (no marking)
3	seminomadic	40	35.3	11.33	
2	Settled	46	47.1	10.64	* (0.05)
4	seminomadic	37	50.2	11.12	
6	Settled	52	47.7	12.00	* (0.01)
5	seminomadic	13	68.7	25.20	
11	Settled	18	65.8	59.4	* (0.01)
12	seminomadic	38	110.0	78.66	

* - p<0.05
Legend as in Figure 1.
